# Lipid bilayer degradation induced by SARS-CoV-2 spike protein as revealed by neutron reflectometry

**DOI:** 10.1038/s41598-021-93996-x

**Published:** 2021-07-21

**Authors:** Alessandra Luchini, Samantha Micciulla, Giacomo Corucci, Krishna Chaithanya Batchu, Andreas Santamaria, Valerie Laux, Tamim Darwish, Robert A. Russell, Michel Thepaut, Isabelle Bally, Franck Fieschi, Giovanna Fragneto

**Affiliations:** 1 Paul Scherrer Institut, Forschungsstrasse 111, 5232 Villigen, Switzerland; 2 Institut Laue-Langevin, 71 Avenue des Martyrs, BP 156, 38042 Grenoble, France; 3 Australian Nuclear Science and Technology Organisation-ANSTO, New Illawarra Rd, Lucas Heights, NSW 2234 Australia; 4 Institut de Biologie Structurale, 71 Avenue des Martyrs, CS 10090, 38044 Grenoble, France

**Keywords:** Biophysics, Structural biology, Chemical physics, Condensed-matter physics

## Abstract

SARS-CoV-2 spike proteins are responsible for the membrane fusion event, which allows the virus to enter the host cell and cause infection. This process starts with the binding of the spike extramembrane domain to the angiotensin-converting enzyme 2 (ACE2), a membrane receptor highly abundant in the lungs. In this study, the extramembrane domain of SARS-CoV-2 Spike (sSpike) was injected on model membranes formed by supported lipid bilayers in presence and absence of the soluble part of receptor ACE2 (sACE2), and the structural features were studied at sub-nanometer level by neutron reflection. In all cases the presence of the protein produced a remarkable degradation of the lipid bilayer. Indeed, both for membranes from synthetic and natural lipids, a significant reduction of the surface coverage was observed. Quartz crystal microbalance measurements showed that lipid extraction starts immediately after sSpike protein injection. All measurements indicate that the presence of proteins induces the removal of membrane lipids, both in the presence and in the absence of ACE2, suggesting that sSpike molecules strongly associate with lipids, and strip them away from the bilayer, via a non-specific interaction. A cooperative effect of sACE2 and sSpike on lipid extraction was also observed.

The coronavirus disease 2019 (COVID-19) is a severe acute respiratory syndrome caused by coronavirus 2 (SARS-CoV-2), a spherical-shaped enveloped virion with a single positive RNA strand within the $\beta$ coronavirus family^1–3^. As with other enveloped viruses, such as the human immunodeficiency virus (HIV), influenza virus and ebola, SARS-CoV-2 exhibits in its envelope a large number of glycosylated proteins, named spike proteins or S-proteins, which are fundamental for the virus to enter the host cell^4,5^. The spike proteins are trimeric class I transmembrane glycoproteins, which recognise the angiotensin-converting enzyme 2 (ACE2), a mammalian transmembrane protein mainly expressed in the lung, intestine, heart, kidney and alveolar epithelial type II cells^3^. Its binding to ACE2 triggers conformational changes that promote the fusion between the host cell membrane and the virus envelope, thereby allowing the viral RNA to be released into the host cell. Compared to other coronaviruses, the spike proteins from SARS-CoV-2 have a very low dissociation constant (14.7 nM) for the binding to ACE2, which makes SARS-CoV-2 highly infectious^6^.

The spike protein is a 180–200 kDa transmembrane protein composed of an N-terminus extracellular domain (ECD), a transmembrane domain (TMD) and a short intracellular domain (ICD)^3,7–9^. It is normally found as a homotrimer and each monomer is composed of two subunits, named S1 and S2. The S1 subunit is mainly located in the outermost part of the ECD and contains the receptor binding domain (RBD) responsible for the interaction with ACE2^3,6,10^. RBD corresponds to residues 319 to 541. The S2 unit includes viral membrane proximal domains as well as the TMD and ICD. The binding of the S1 unit to ACE2 via the RBD is fundamental to convert the S2 unit from a metastable pre-fusion state into a more stable post-fusion state, which allows the virus envelope to fuse with the plasma membrane of the host cell^11^.

The extracellular and soluble domain of the spike protein (hereafter referred to as *sSpike*) is being intensively studied as a target for vaccines^8,9,12,13^. So far, these studies focused on the structure of sSpike alone or in complex with ACE2. However, a detailed structural description of the potential interactions of the sSpike with lipids in the mammalian plasma membrane is currently missing. This is a particularly relevant aspect since ACE2 is only the first target of interaction for the spike proteins and subsequent or parallel interactions with the surrounding membrane lipids are also pivotal for membrane fusion and viral entry in the host cell^1,3^. In order to deepen the current knowledge on the complex virus-host cell interaction, we have investigated the interaction between the sSpike from SARS-CoV-2 and supported lipid bilayers (SLBs) of different compositions, from simple to more complex mimics of the mammalian plasma membrane. The sSpike comprises residues 1 to 1208 of total spike (1272 residues in total) and exhibits a structural conformation corresponding to the pre-fusion state (fully-opened conformation)^14^. The characterization of the sSpike-membrane interaction was performed both in the presence and absence of the ACE2 extracellular domain (hereafter referred to as *sACE2*), aiming at highlighting the impact of the spike-ACE2 interactions on membrane structure and stability.

In this study, synthetic SLBs composed of 1-palmitoyl-2-oleoyl-glycero-3-phosphocholine (hPOPC) and 1-palmitoyl-2-oleoyl-sn-glycero-3-phospho-L-serine (hPOPS) (70:30 mol/mol) were used as a reference lipids. Indeed, PC and PS lipids are among the most abundant lipids in the mammalian plasma membrane^15^ and the structure of POPC/POPS SLBs is well documented^16–19^. We had no access to the fully deuterated forms of these lipids. In order to investigate a more biologically relevant lipid system, we also produced SLBs with a mixture of deuterated natural phospholipids extracted from the yeast *Picha pastoris* and deuterated cholesterol (10 %_mol_). The natural phospholipid mixture contained phosphatidylcholine (PC), phosphatidylserine (PS) and phosphatidylethanolamine (PE) at the molar ratios PC:PS:PE:chol 59:20:11:10. The natural phospholipid mixtures were characterised by a polydisperse acyl chain length composition (C16-18) and a certain level of unsaturation (1–3 double bonds)^20,21^. Although mammalian plasma membranes exhibit an asymmetric composition of the inner and outer bilayer leaflet, given the overall abundance of such lipid components and cholesterol in those membranes^15^, they were chosen in our study to reproduce biologically relevant models^16^.

The produced SLBs and their interaction with the sSpike protein were studied by neutron reflectometry (NR). NR is a widely used technique in the field of membrane biophysics and allows to probe the bilayer structure with a resolution of the order of few Å  in the direction perpendicular to the membrane surface^22^. In addition, due to the different interactions of neutrons with the chemical groups present in proteins and lipids, quantitatively expressed by the corresponding scattering length density, NR allows to distinguish the lipid membrane from potentially attached or penetrating protein molecules^23^. In this context, the use of deuterated lipids is particularly relevant for increasing the difference in scattering length density (neutron contrast) between lipids and proteins^24^. Therefore, to obtain the deuterated lipid mixtures, *P. pastoris* cells were grown in a deuterated culture medium^20,21,25,26^.

The interpretation of the NR data was supported by complementary quartz crystal microbalance with dissipation monitoring (QCM-D) experiments. This technique is a well established tool for the study of adsorption of (macro)molecules onto solid substrates, including the analysis of viscoelastic film properties^27,28^.

SLBs were prepared according to a recently reported method based on the fusion of so called *peptide discs* onto a solid surface^17,29^. Peptide discs consist of discoidal lipid bilayers of about 10 nm diameter, stabilised in solution by a belt of self assembled 18A peptide molecules^30^. Upon deposition of the peptide disc solution onto a solid substrate, the peptide molecules can be removed by buffer rinsing, leaving a supported lipid bilayer on the surface. This preparation method was recently reported as particularly efficient to prepare SLBs composed of complex lipid mixtures, such as natural lipid extracts or embedded transmembrane proteins^17,29^

Altogether, the collected data suggest that the injection of sSpike molecules both in the presence and in the absence of ACE2 produces a significant degradation of the SLB, as indicated by the large reduction of the surface coverage. A similar behaviour was observed both in the case of the simple synthetic hPOPC/hPOPS bilayer as well as in the case of the more complex mixture mimicking a deuterated plasma membrane (hereafter referred to as *dPM*). This result suggests that besides the high affinity for ACE2, the spike protein also exhibits a strong association with membrane lipids, which might play an important role in the membrane fusion event that allows the virus to enter the host cells. Understanding the interaction between sSpike and membrane lipids might provide new insight on the mechanism of viral infection, and it might offer input for the design of new therapeutic strategies.

## Results

**Figure 1:**
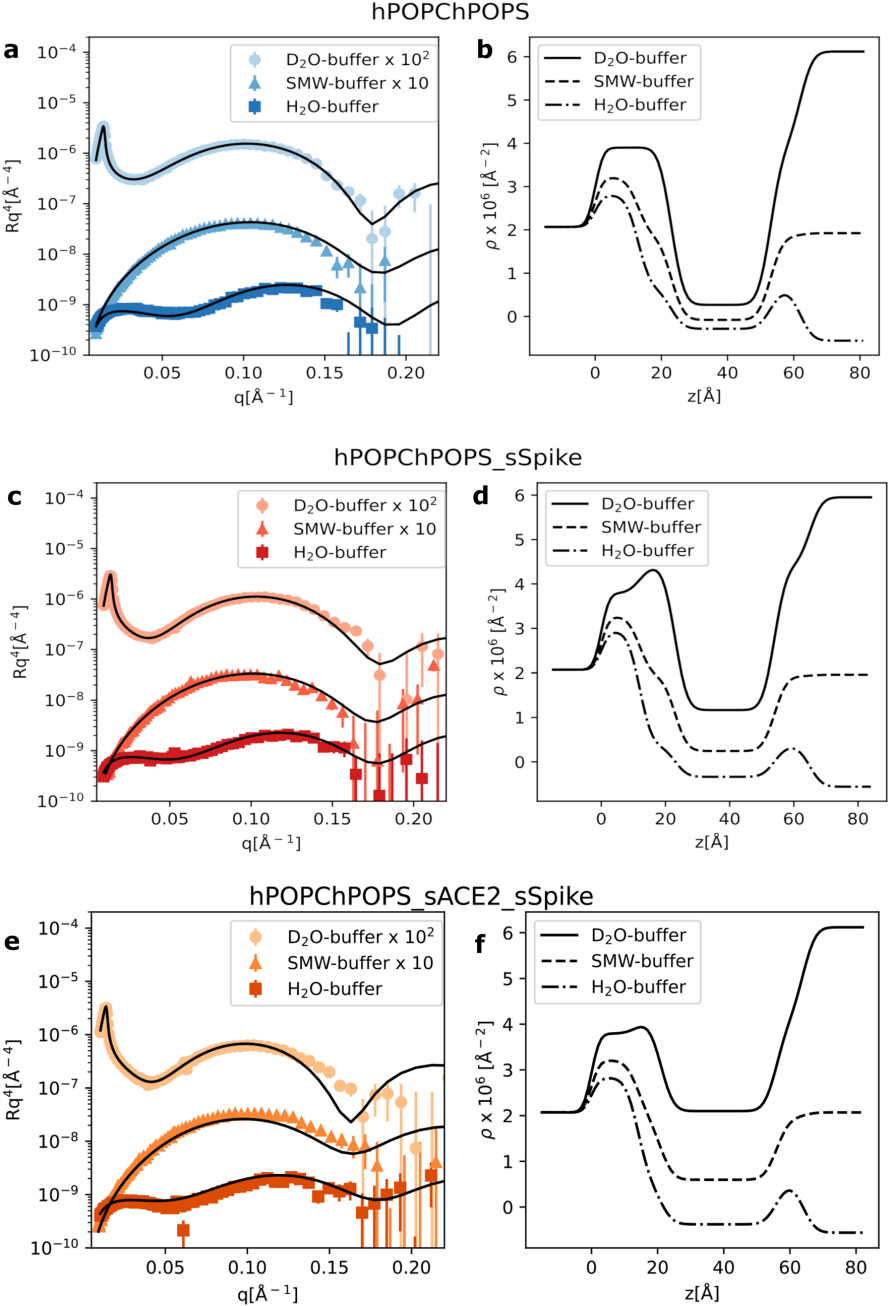
NR experimental data together with the fitting curves from a hPOPC/hPOPS lipid bilayer (**a**) before, (**c**) after injection of the sSpike solution, (**e**) after sequential injection of sACE2 and sSPike, and (**b**,**d**,**e**) corresponding scattering length density profiles calculated from NR data analysis. Data and fits are scaled for clarity as reported in the legend.

We initially investigated the impact of sSpike on the structure and stability of a hydrogenous SLB prepared from a synthetic hPOPC:hPOPS (70:30 mol/mol) lipid mixture. Figure 1 shows the NR data with the corresponding best fit curves before (a) and after (c) the injection of the sSpike solution (0.468 mg/mL) and the corresponding scattering length density profiles ($\rho (z)$) obtained from data analysis. The reflectivity of each sample was measured in three different contrasts (see “Methods” section).

In the case of pure hPOPC/hPOPS SLB, the theoretical model matches well to the experimental data. Overall, the structure of the bilayer (Table 1) is, as expected, comparable with previously reported results^17^. Upon injection and equilibration (30 min) of the sSpike, the surface was rinsed with buffer to remove excess protein. A change in the experimental data was observed after protein injection, which was initially ascribed to sSpike-bilayer association. A theoretical model including a lipid bilayer and a protein layer at the bilayer surface was tested. Indeed, the scattering length density of the bilayer was unaffected by the protein injection, indicating that there is no protein inside the lipid acyl chains. The optimisation of the protein layer parameters suggested a very large solvent content (i.e. $\sim 0.99$), which is consistent with either the complete absence of sSpike molecules on the bilayer surface or a sSpike concentration at the bilayer surface below the NR detection limit (a few percent). For this reason, in the final data analysis we used a model involving only the presence of the lipid bilayer, since it is still possible to determine the extent of protein/lipid interaction by looking at the variation of the bilayer structure upon interaction with the sSpike. Data analysis indicated that although no significant amount of sSpike was associated to the SLB, the bilayer structure varied upon protein injection (Table 1). More specifically, a considerable decrease of the surface coverage, indicated by a large increment of 0.1 v/v solvent volume fraction among the lipid acyl chains (i.e. 0.13 v/v and 0.23 v/v before and after protein injection, respectively), was observed. Typically, SLBs are stable when rinsed with buffer solution and only a small variation of surface coverage ($\le$ 0.05 v/v) is observed. Therefore, the reported coverage reduction cannot be simply ascribed to the process of rinsing during contrast variation measurements, but is more likely the result of the interaction between the sSpike and the lipid bilayer.

**Table 1:** Optimised parameters from NR data analysis: thickness (*t*), solvent volume fraction ($\phi _s$), scattering length density ($\rho$).

hPOPC/hPOPS	Acyl chains	Headgroups
*t* (Å)	$30 \pm 2$	$9 \pm 1$
$\phi _s$	$0.13 \pm 0.06$	$0.49 \pm 0.05$
$\rho \cdot 10^{-6}$ (Å$^{-2}$)	$-0.27 \pm 0.03$	$1.77 \pm 0.02$
hPOPC/hPOPS+sSpike	Acyl chains	Headgroups
*t* (Å)	$31 \pm 1$	$12 \pm 2$
$\phi _s$	$0.23 \pm 0.03$	$0.62 \pm 0.01$
$\rho \cdot 10^{-6}$ (Å$^{-2}$)	$-0.28 \pm 0.03$	$1.86 \pm 0.02$
hPOPC/hPOPS+ACE2	Acyl chains	Headgroups
*t* (Å)	$32 \pm 1$	$8 \pm 2$
$\phi _s$	$0.12 \pm 0.04$	$0.39 \pm 0.01$
$\rho \cdot 10^{-6}$ (Å$^{-2}$)	$-0.29 \pm 0.02$	$1.88 \pm 0.02$
hPOPC/hPOPS+sACE2+sSpike	Acyl chains	Headgroups
*t* (Å)	$32 \pm 2$	$7\pm 2$
$\phi _s$	$0.35 \pm 0.06$	$0.49 \pm 0.02$
$\rho \cdot 10^{-6}$ (Å$^{-2}$)	$-0.29 \pm 0.02$	$1.9 \pm 0.1$
dPM	Acyl chains	Headgroups
*t* (Å)	$34 \pm 3$	$10\pm 1$
$\phi _s$	$0.11 \pm 0.01$	$0.52 \pm 0.05$
$\rho \cdot 10^{-6}$ (Å$^{-2}$)	$5.13 \pm 0.04$	$6.8 \pm 0.2$
dPM+sACE2+sSpike	Acyl chains	Headgroups
*t* (Å)	$31 \pm 4$	$9\pm 2$
$\phi _s$	$0.53 \pm 0.01$	$0.75 \pm 0.04$
$\rho \cdot 10^{-6}$ (Å$^{-2}$)	$5.0 \pm 0.4$	$6.7 \pm 0.1$
dPM+RDB	Acyl chains	Headgroups
*t* (Å)	$35 \pm 3$	$10\pm 2$
$\phi _s$	$0.19 \pm 0.02$	$0.5 \pm 0.1$
$\rho \cdot 10^{-6}$ ( Å$^{-2}$ )	$5.3 \pm 0.1$	$6.5 \pm 0.7$

Surface roughness was in the range of 3–4 *Å* for all the investigated samples.

Similarly to the experiment described above, NR data were collected on a hPOPC:hPOPS bilayer formed in presence of sACE2 molecules (further details are reported in the Method section and Supplementary Material). Briefly, the peptide discs were either suspended in sACE2 solution and then purified by size exclusion chromatography, or the lipid bilayer was formed and then exposed to the sACE2 solution (incubation time 30–60 min). During both preparation procedures, the sACE2 molecules interacted with the lipid headgroups and most likely associated with the bilayer surface, as there was no evidence of their direct adsorption onto the solid surface with consequent formation of a highly hydrated underneath layer. Unfortunately, in both cases the amount of embedded protein was below the detection limit of NR, and it was not possible to quantify the sACE2 on the SLB surface (Table 1).The hPOPC/hPOPS bilayer produced with and without sACE2 exhibited comparable structural parameters, which indicates that sACE2 does not affect the bilayer structure. On the other hand, there was clear evidence of a different behavior of the sSpike/bilayer system in presence of sACE2: upon sSpike injection, a more pronounced bilayer destabilisation compared to the pure hPOPC:hPOPS was observed (Fig. 1e,f), with the solvent volume fraction in the lipid acyl chains increasing from $0.12\pm 0.04$ v/v to $0.35\pm 0.06$ v/v after sSpike injection. This result suggests a cooperative effect of sACE2/sSpike on the lipid extraction.

**Figure 2:**
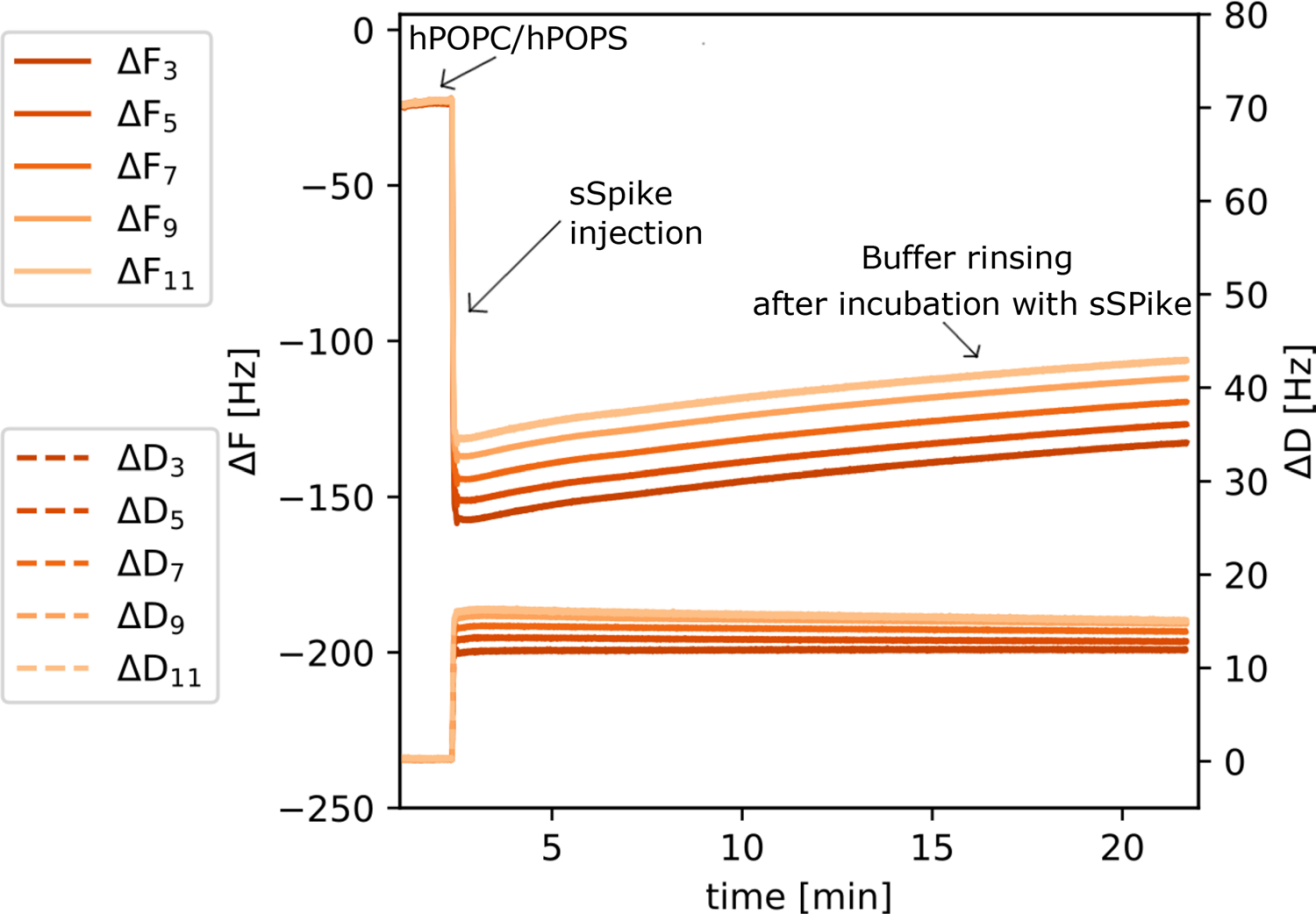
QCM-D data collected during the injection of sSpike solution (0.468 mg/mL) on a supported lipid bilayer from hPOPC:hPOPS:sACE2. The supported bilayer was prepared by deposition of hPOPC/hPOPS peptide discs, previously incubated with sACE2 (0.1 mg/mL).

The lipid stripping from a previously formed bilayer was confirmed by the QCM-D measurements. Figure 2 shows QCM-D data collected during formation of a hPOPC:hPOPS SLB followed by addition of sSpike and buffer rinsing (see also additional data in SM). A $\Delta F$ of approximately − 25 Hz was initially recorded, a typical value for a synthetic SLB on the QCM-D sensor. Upon injection of the sSpike solution (0.5 mL at 0.468 mg/mL), a significant decrease of $\Delta F$ (in the range − 130 Hz/− 160 Hz) and consequent increase of $\Delta D$ (in the range 12 Hz/− 16 Hz) was recorded. In addition the $\Delta F$ and $\Delta D$ corresponding to the different sensor overtones are no longer well superimposed, which indicates the formation of a highly viscoelastic layer. Altogether, these results are consistent with the adsorption of highly hydrated sSpike molecules on the bilayer. The lack of evidence in the NR data of adsorbed protein molecules on the SLB, together with the large $\Delta F$ variation upon sSpike injection, supports the hypothesis of the presence of a highly diluted protein layer at the SLB surface. Interestingly, the adsorption of sSpike is immediately followed by a slow but constant decrease of coupled mass on the sensor surface (increase of $\Delta F$ to − 135 Hz/− 115 Hz and decrease of $\Delta F$ to 11 Hz/15 Hz), in good agreement with the interpretation of the bilayer degradation from the NR analysis. However, the stripping process does not produce a clear increment of dissipation, which can be explained by the homogeneous ’dilution’ of the lipids all along the surface, rather than by the formation of water cavities of highly viscous behaviour. The most probable mechanism elaborated from the data interpretation is the bilayer degradation produced by lipid stripping is not a consequence of buffer rinsing, as it starts immediately upon sSpike/lipid contact. In addition, such a layer degradation has been reported also for other model lipid bilayers as a consequence of the interaction with the sSpike protein^31^.

**Figure 3:**
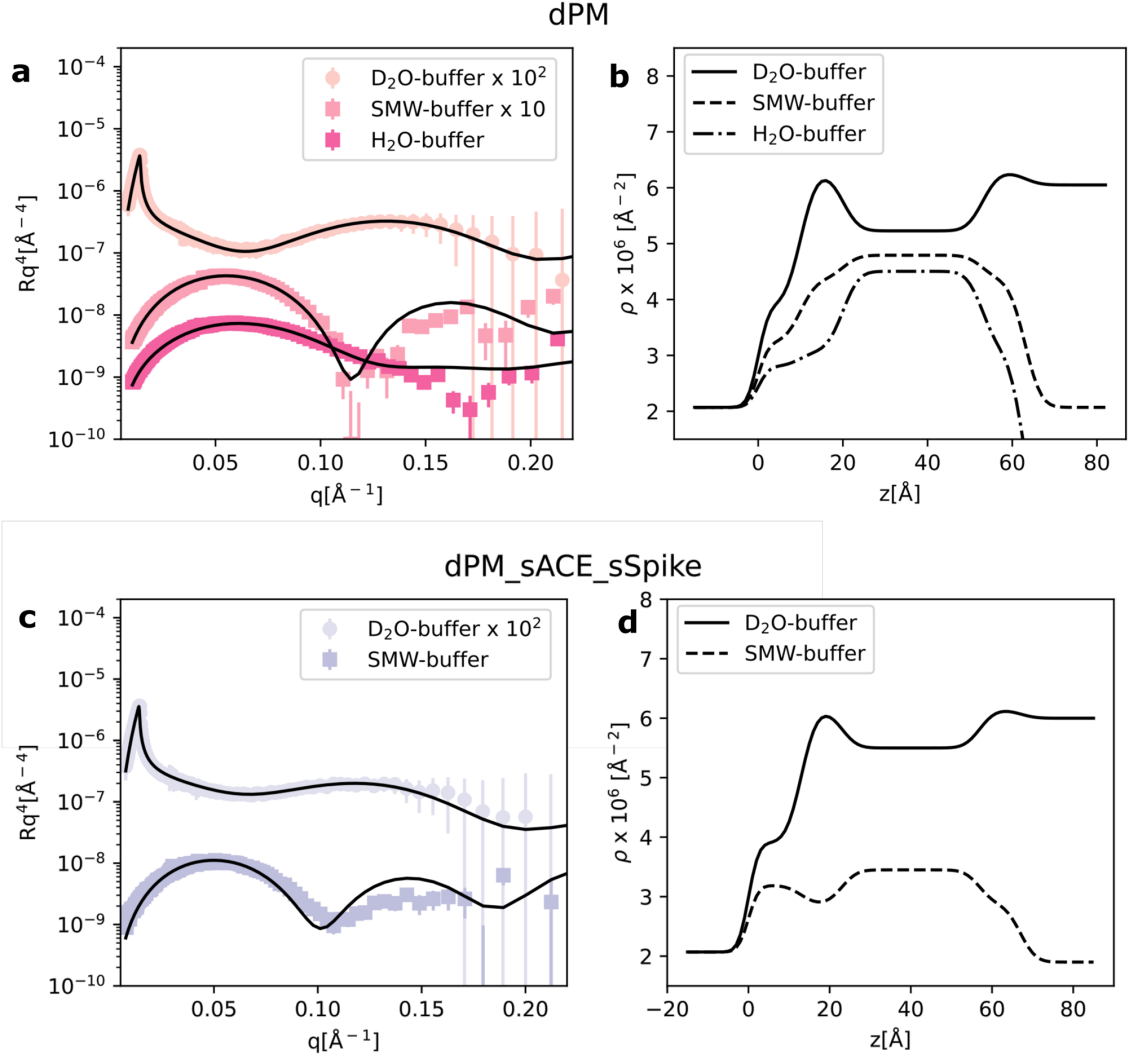
NR experimental data together with the fitting curves from dPM lipid bilayer samples before (**a**) and after sequential injection of sACE2 and sSpike solutions (**c**). Scattering length density profiles determined by NR data analysis (**b**,**d**). Data and fits are scaled for clarity as reported in the legend.

The combined effect of sACE2 and sSpike was also investigated on dPM, a more biologically relevant system, prepared from a mixture of natural phospholipids, extracted from the yeast *P. pastoris*, and cholesterol. The phospholipid species used were PC:PS:PE with molar ratios 59:11:20 in the presence of 10%_mol_ cholesterol. To enhance the contrast between lipids and proteins, the lipids were perdeuterated. Figure 3a and b show the NR experimental data, the best fit and the corresponding scattering length density profiles. Data analysis confirmed the successful formation of a SLB even with this complex lipid composition. Thicker bilayers in comparison to the synthetic SLBs were formed, according to our expectation based on the varying acyl chain composition and the presence of cholesterol. The estimated scattering length density of  the headgroup layer ($(6.8\pm 0.2)\cdot 10^{-6}$ Å$^{-2}$) is in good agreement with the calculated theoretical value for the dPC, dPE, dPS headgroups ($7.2\cdot 10^{-6}$ Å$^{-2}$), while a lower scattering length density ($(5.13\pm 0.04)\cdot 10^{-6}$ Å$^{-2}$) was found for the acyl chain region including 10%mol of deuterated cholesterol compared to the calculated value (i.e. $6.9\cdot 10^{-6}$ Å$^{-2}$). This evidence suggests that some hydrogenous contaminant, most likely introduced during the SLB preparation, is retained within the lipid acyl chains (e.g. detergent from protein solutions or residual peptide molecules). Such contaminant traces did not have any impact on the SLB structure, which was comparable to previously reported results^17,32,33^, and are not expected to have an impact on the interaction between the SLB and ACE2/sSpike, the latter involving the headgroup region only.

Upon sequential injection of the sACE2 and sSpike solutions, a similar effect as the one observed for the synthetic SLBs was found (Fig. 3c,d), namely the reduction of the surface coverage. This is deduced from the increment of the solvent volume fraction in the acyl chains, with 0.42 v/v for dPM and 0.2 v/v for hPOPC/hPOPS bilayers.

## Discussion

The spike protein is located in the envelope of coronaviruses, such as SARS-CoV-2, and has a central role in the membrane fusion events that allow the virus to enter the host cell^3^. Because of this fundamental function, the spike protein is the focus of current research for the development of efficient vaccines against COVID-19^11^. These are mostly based on antibodies targeting the spike proteins, therefore preventing binding to ACE2. However, several mutations of the spike sequence are rapidly emerging^34^, which can strongly affect the efficacy of the already developed vaccines.

**Figure 4:**
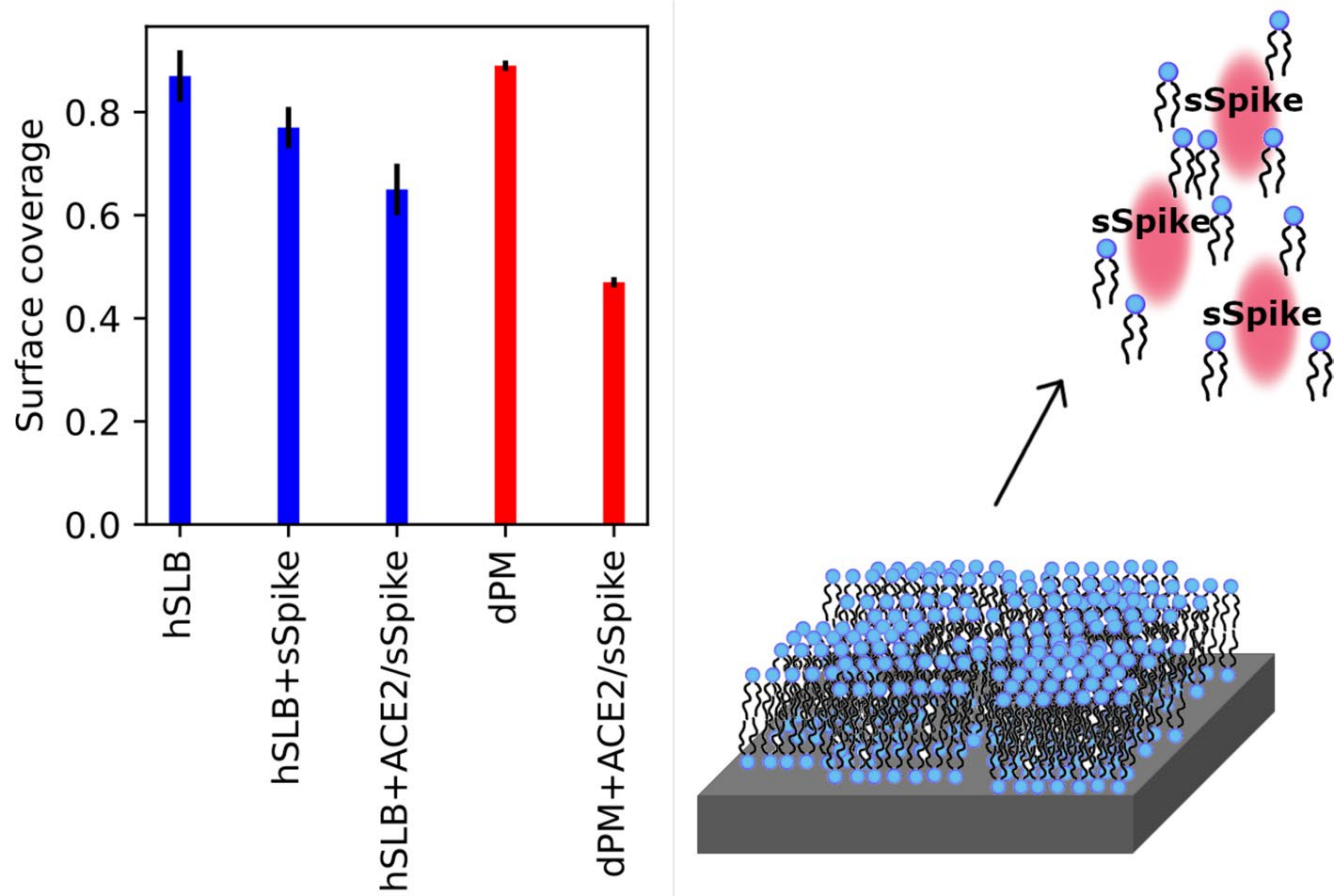
Bilayer surface coverage before and after the addition of sSpike to the hydrogenous synthetic SLB, i.e. hPOPC/hPOPS and the deuterated natural SLB, i.e. dPM. Schematic representation of the bilayer degradation produced by the sSpike.

Detailed information on the interaction between the spike proteins and the mammalian plasma membrane can provide useful insight on the membrane fusion process and potentially help with identifying new strategies for preventing the infection. To contribute to the strengthening of the knowledge in this research area, we built simple, but biologically relevant, model systems consisting of supported lipid bilayers composed of synthetic or natural lipid mixtures, and we tested their interaction with the soluble, extramembrane domain of the spike protein in the presence and in the absence of the extracellular domain of ACE2 (sACE2). The main outcome of our studies was that the injection of sSpike, both on a bare SLB or after incubation of the SLB with sACE2, produced a remarkable degradation of the lipid bilayer, resulting in a reduction of the bilayer surface coverage, as summarised in Fig. 4. The observed coverage reduction suggests that sSpike molecules might strongly associate with lipids, which are stripped away from the surface immediately after sSpike protein injection, in the presence and in the absence of sACE2. We intend to further investigate this aspect in future experiments on more complex membrane model systems and by incorporating full-length ACE2 in the SLB. Lipid binding pockets were previously identified in the sSpike structure and located close to the RDB^35–38^. In particular, the association of free fatty acids such as linoleic acid, is believed to stabilise the closed form of the sSpike^35^. Therefore, an hypothesis which could explain the observed degradation is that the same lipid binding pockets come from the stripping of unsaturated lipids from the membrane. This process might favour the transition of the spike protein from the open to the close conformation. Indeed, a conformational change of the sSpike upon binding to ACE2 was reported as crucial for bringing membrane proximal region of the S1 unit close to the plasma membrane host cell^3,14^. To verify the validity of this assumption similar NR experiments on a natural SLB, dPM, were carried out using the receptor binding domain only at a concentration of 0.92 mg/mL (see Supplementary Materials). The variation of surface coverage before and after the sequential addition of sACE2 and RDB was approximately 0.15 v/v, much smaller than the variation of surface coverage observed when the full sSpike was used. It is worth noting here﻿ that only the extracellular domain of the SARS-CoV-2 spike protein is used in our studies. Despite the RDB, this protein domain contains also part of the sequence of the S2 subunit, which is believed to be involved in the membrane binding and membrane fusion process^3^, as for the SARS-CoV virus. Our results support the hypothesis of the presence in the sSpike sequence of various membrane binding sites, which are not limited to the bare interaction between RDB and ACE2, but which might also imply more complex rearrangements of the protein structure by lipid binding.

## Methods

### Chemicals

The deuterated lipid extract was obtained from *P. Pastoris* yeast cells, GS115 (his4) grown in D$_2$O media (further details are reported in the next subsection). The deuterated cholesterol (d-45 cholesterol, 85% deuteration) was provided by the National Deuteration Facility at ANSTO (Sydney, Australia). The details of the biosynthetic production of d-45 Cholesterol are reported elsewhere^39^. The 18A peptide (sequence KAFYDKVAEKLKEAF acetylated and amidated at the N-terminal and C-terminal, respectively, >95% purity) was obtained from GenScript. Heavy water (D$_2$O 99.9% purity), chloroform (>99.5% purity), ethanol (98% purity), and methanol (99.8% purity) were from Sigma-Aldrich. All purchased chemicals were used as received, without further purification. The extracellular domain (740aa) of ACE2, sACE2, was purchased from Synobiological.

### sSpike expression and purification

Spike HexaPro was expressed in EXPI293 cells and purified, as described previously^40^. Finam gel filtration buffers contained 25 mM Tris pH 8, 150 mM NaCl. Concentrated sample was at 0.468 mg/mL. SARS-CoV-2 Spike RBD domain was expressed and purified as follows. The mammalian expression vector used for the RBD production, was vector NR-52309 from BEI Resources. EXPI293 cells grown in EXPI293 expression medium were transiently transfected with the vector according to the manufacturer’s protocol (Thermo Fisher Scientific). Cultures were harvested five days after transfection and the medium was separated from the cells by centrifugation. Supernatant was passed through a 0.45 μm filter and used for a two-step protein purification on Äkta Xpress, with a HisTrap HP column (GE Healthcare) and a Superdex 75 column (GE Healthcare). Before sample loading, columns were equilibrated in 20 mM Tris pH 7.5 and 150 mM NaCl buffer. Unbound proteins were eluted from the affinity column with equilibration buffer, contaminants with the same buffer supplemented with 75 mM imidazole while the RBD was eluted with equilibration buffer supplemented with 500 mM imidazole and immediately loaded onto the gel filtration column run in equilibration buffer. Fractions of interest were pooled and concentrated at 0.92 mg/mL on an Amicon Ultra 10 K centrifugal filter according to the manufacturer’s protocol (Millipore). The concentration of purified protein was estimated using an absorption coefficient at 280 nm of 33850 M-1 cm-1 calculated using the PROTPARAM program (http://web.expasy.org/protparam/) on the Expasy Server.

### *Pichia pastoris* cell cultures

*Pichia pastoris* cells were cultured as reported previously in the Deuteration facility of the Institut Laue-Langevin (D-Lab, ILL) Grenoble, France^25,32^. Cells were grown in flasks at 30$^{\circ }$C using a basal salt medium (BSM) as the minimal medium at pH 6.0 (*P. pastoris* fermentation process guidelines, Invitrogen, United States) containing either 20 g/L of glycerol in H$_2$O or glycerol-d8 (Euriso-Top, France) in D$_2$O. Cells upon entering the exponential phase at an OD of 600 were harvested by centrifugation and frozen at $-80^{\circ }$C.

### Natural lipid extraction and purification

Biomimetic membranes were prepared from phospholipids extracted and purified from perdeuterated and hydrogenous *P. pastoris* biomass^17,20,25,26^. Harvested cells were suspended into 10 mL deionised water and lysed by probe sonication on an ice bath for 35 min with 30 s intervals, 25% duty cycle. The resulting cell lysate was poured into boiling ethanol containing 1% butylated hydroxytoluene (BHT) followed by vigorous stirring in order to denature lipases that have the ability to hydrolyse phospholipids. Lipids were then extracted according to the method of Folch et al.^41^, followed by evaporation of the organic phase under a N$_2$ stream and their final reconstitution in CHCl$_3$. Purification of the various classes of phospholipid mixtures containing molecular species of mixed acyl chain lengths was achieved through sequential purifications first through an amino-bonded solid-phase extraction column followed by a diol-modified silica stationary phase column coupled to a High-Performance Liquid Chromatography-Evaporative light scattering detector (HPLC-ELSD) [Agilent 1260, United Kingdom] system. The mobile phase employed was a gradient of solvent A (CHCl$_3$/CH$_3$OH/NH$_4$OH, 80:20.5:0.5, v/v) and solvent B (CHCl$_3$/CH$_3$OH/H$_2$O/NH$_4$OH, 60:35:5.5:0.5, v/v) (Boselli et al. 2012). TLC analysis was carried out on a High-Performance Thin-Layer Chromatography (HPTLC) system (CAMAG, Muttenz, Switzerland) to assess the identity and purity of each of the purified classes. Fatty acid compositions of such purified mixtures were measured by Gas Chromatography-Flame Ionization Detection (GC-FID).

### Supported lipid bilayer preparation

Supported lipid bilayers were prepared by deposition of peptide discs and subsequent peptide removal by rinsing. According to the experimental procedure reported previously^30^, peptide discs with synthetic and natural lipids were prepared by mixing lipids and 18A peptide (1:1 w/w) in a chloroform/methanol mixture. Subsequently the organic solvents were removed under a nitrogen stream to produce a dried film of mixed lipids and 18A peptide. The film was suspended in 20 mM Trizma, 100mM NaCl and 3mM CaCl$_2$ buffer solution at pH 7.4 (lipid:buffer 1:1 w/w) and the suspension was purified by size exclusion chromatography (SEC) with a Superdex 200 10/300 GL increase column to separate the peptide discs from other aggregates. The collected fractions (2 ml in total) corresponding to the peptide disc elution peak were stored at $-20^{\circ }$C until use.

SLBs were prepared in-situ by injection of 2 mL of peptide disc solution on a monocrystalline silicon substrate (80 mm $\times$ 50 mm $\times$ 15 mm), previously sealed inside a cell designed for the NR experiments at the solid/liquid interface. The peptide discs were incubated for 20 min and rinsed by buffer solution (20 mL at 1 mL/min) to promote the disc fusion and the removal of the peptide belt. All measurements were carried out at controlled temperature of $25^{\circ }$C with a thermal circulating water bath.

### Neutron reflectometry

Neutron Reflectometry (NR) experiments were performed at the D17^42^ and FIGARO^43^ reflectometers at the ILL (doi:10.5291/ILL-DATA.DIR-207). Both instruments operate in time of flight mode, with a horizontal scattering plane for D17 and a vertical scattering plane for FIGARO. Two incoming angles were used of 0.8$^{\circ }$ and 3$^{\circ }$ on D17 and 0.8$^{\circ }$ and 3.2$^{\circ }$ on FIGARO, to cover a q-range of about 0.003Å$^{-1}$ − 0.25 Å$^{-1}$, with a wavelength range between 2 and 20Å on FIGARO and 2 and 25Å on D17, the upper q limit being set by the sample background signal. The background was measured on the left and right sides of the reflectivity signal and subtracted from the measured reflected intensity. The reflected intensity was normalised to the direct beam measured at the same instrumental configurations, the slit settings were selected to provide a constant surface under-illumination at the different angles of incidence.

NR data were analysed with an in-house developed code that is based on the Parratt formalism to calculate the reflectivity profile corresponding to a given structural model^44^. A standard approach in NR data analysis is to consider the sample as composed by a stack of layers, each of them characterised by a different thickness (*t*), scattering length density (${\rho }$), solvent volume fraction (${\phi }_{s}$) and surface roughness ($\sigma$).The scattering length density is defined as

1 $$\begin{aligned} \rho =\sum _{i}\frac{n_i b_i}{V_m} \end{aligned}$$

where $n_i$ is the number of i-type nuclei and $b_i$ is the corresponding coherent scattering length, while $V_m$ is the molecular volume. Further details about the NR data analysis procedure are reported in supplementary materials.

The investigated SLBs were modelled as a stack of four layers: (1) silicon oxide, which spontaneously forms on the surface of crystalline silicon substrates; (2) inner lipid headgroups in the proximity of the support surface; (3) lipid acyl chains belonging to the two bilayer leaflets; (4) outer lipid headgroups in contact with the bulk solvent. A fifth layer was included in the model for the bilayer exposed to protein solution in order to take into account those molecules adsorbed on the bilayer surface. The structural parameters associated with layer 1 were obtained from the analysis of independent datasets collected for the silicon substrate in D$_2$O and H$_2$O contrasts. All the parameters of layer 1, except the solvent volume fraction, were kept fixed during the data analysis. A preliminary analysis revealed that all the investigated samples are characterised by the same composition of the inner and outer leaflet, and confirmed their structural symmetry. For this reason, in the final data analysis layer 2 and 4 were constrained to have the same parameter values.

NR measurements were performed in different contrasts with the following compositions: D$_2$O buffer ($\rho =6.35\cdot 10^{-6}$Å$^{-2}$), 4-matched-buffer (4MW-buffer 66:34 D$_2$:OH$_2$O v/v, $\rho =4\cdot 10^{-6}$Å$^{-2}$), silicon-matched-buffer (SMW-buffer 38:62 D$_2$O:H$_2$O v/v, $\rho =2.07\cdot 10^{-6}$Å$^{-2}$) and H$_2$O buffer ($\rho =-0.56\cdot 10^{-6}$Å$^{-2}$). Data collected in the different contrasts were simultaneously analysed with the same structural model.

### Quartz crystal microbalance with dissipation monitoring

QCM-D measurements were performed with the same protocol as previously reported^29,45,46^ with a E4 instrument (Q-Sense, Biolin Scientific AB, Sweden), using SiO$_2$-coated 5 MHz quartz sensors in the PSCM labs at the ILL. Crystals and O-rings were cleaned in Hellmanex 2% aqueous solution for 20 min, extensively flushed with ethanol and ultrapure water, and then dried under a nitrogen flow. Immediately before use, the crystals were treated with a UV ozone cleaner (BioForce Nanosciences, Inc., Ames, IA) for 30 min. The fundamental frequency and six higher overtones (3rd, 5th, 7th, 9th, 11th and 13th) were recorded in ultrapure water (Milli-Q 18 M$\Omega$ cm at 25C, Millipore) until a stable baseline was obtained. After exchange with buffer, the samples were injected in the flow cell at 0.2 mL/min, and the real-time shifts in the resonance frequency ($\Delta F_n$) with respect to the calibration value (bare crystal in buffer) were measured for different overtones indicated as $F_n$, with *n* representing the overtone number. Simultaneously, also the energy dissipation factor (*D*) was monitored for all the measured overtones.

## Supplementary Information

Supplementary Information 1.
