# Supplementary material for: Lipid bilayer degradation induced by SARS-CoV-2 spike protein as revealed by neutron reflectometry
Source: Sci Rep. 2021 Jul 21;11:14867. doi: 10.1038/s41598-021-93996-x (PMC8295359; doi:10.1038/s41598-021-93996-x)
Supplement: Supplementary file 1 — Supplementary Information 1. [file 41598_2021_93996_MOESM1_ESM.pdf]

Lipid bilayer degradation induced by SARS-CoV-2 Spike protein as revealed by neutron reflectometry
Supplementary Material

Alessandra Luchini, [a,+], Samantha Micciulla,[b,+] Giacomo Corucci,[b]
Krishna Chaithanya Batchu,[b] Andreas Santamaria, [b]Valerie Laux,[b]
Tamim Darwish,[c] Robert A. Russel, [c] Michel Thepaut, [d]
Isabelle Bally, [d] Franck Fieschi, [d] and Giovanna Fragneto [b]

[a] *Paul Scherrer Institut, Forschungsstrasse 111, 5232 Villigen, Switzerland*
[b] *Institut Laue-Langevin, 71 Avenue des Martyrs, BP 156, 38042 Grenoble, France*
[c] *Australian Nuclear Science and Technology Organisation-ANSTO, New Illawarra Rd, NSW 2234, Lucas Heights, Australia*
[d] *Institut de Biologie Structurale, 71 avenue des Martyrs, CS 10090, 38044, Grenoble, France*
* fragneto@ill.eu
+ these authors contributed equally to this work

1 Neutron Reflectometry (NR) data analysis

NR data were analysed with an in-house developed code which is based on the Parratt formalism to calculate the reflectivity profile corresponding to a given structural model [1]. A standard approach in NR data analysis is to consider the sample as composed by a stack of layers, each of them characterised by a different thickness (t), scattering length density (ρ), solvent volume fraction (ϕ_s) and surface roughness (σ).The scattering length density

is defined as

$$\rho = \sum_i \frac{n_i b_i}{V_m} \tag{1}$$

where n_i is the number of i-type nuclei and b_i is the corresponding coherent scattering length, while V_m is the molecular volume.

In the code used to analyse the NR data, the sample layers are numbered from 1 to $N-1$, where index 0 identifies the medium from which the neutrons enter the sample, and where the reflectivity is measured, and N is the buffer solution on the other end of the sample. In the performed experiments, medium 0 corresponds to the silicon support. The above listed layer parameters are used to calculate the refractive index associated to each layer according the Parratt's recursive formula [1]. More specifically, for a generic layer i in the sample, the refractive index (r_i) is calculated as

$$r_i = \frac{f_i + r_{i+1} e^{q_i t_i} e^{0.5 q_i q_{i+1} \sigma_i}}{1 + f_i r_{i+1} e^{q_i t_i} e^{0.5 q_i q_{i+1} \sigma_i}} \tag{2}$$

where

$$f_i = \frac{q_i - q_{i-1}}{q_i + q_{i+1}} \tag{3}$$

q_i, q_{i+1}, q_{i-1} are the scattering vectors in the i, $i+1$ and $i-1$ layers and t_i and σ_i are the thickness and the roughness of the i-th layer. ρ_i is used to calculated the scattering vector in each layer according to

$$q_i = \sqrt{q_0^2 + 16\pi(\rho_0 - \rho_i)} \tag{4}$$

where q_0 is the measured q in the experimental data and ρ_0 is the scattering length density of the support.

The refractive index at the interface between the sample and the buffer is calculated from equation 3, while the refractive index of the layers below the interface with the buffer are calculated according to equation 2 from layer $N-1$ to 0. The theoretical reflectivity (R) is the square modulus of the refractive index at interface 0, the interface between the sample and support.

The experimental data are characterised by a calculated q-resolution ($\frac{\Delta q}{q}$) which is provided both at SURF and D17. While the data collected on SURF are characterised by a constant $\frac{\Delta q}{q} = 0.05$, the data collected on D17 have a q-dependent $\frac{\Delta q}{q}$ in the range 0.036-0.10. This q-resolution is taken into account in the theoretical model by convoluting the calculated reflectivity with a Gaussian function of standard deviation $2\sqrt{2log2\frac{\Delta q}{q}}$.

Optimisation of the model parameters (t, ρ, ϕ_s, σ for each layer) produces the reflectivity

theoretical curve that better resembles the experimental data. A scattering length density profile ($\rho(z)$) is also calculated from the optimised parameters, which graphically represents how the different sample layers are distributed in the direction perpendicular to the substrate surface (z). Errors on the optimised parameters are calculated as the square-root of each of the diagonal elements of the covariance matrix, which is multiplied by the reduced χ^2.

2 hPOPC/hPOPS SLB after injection of ACE2

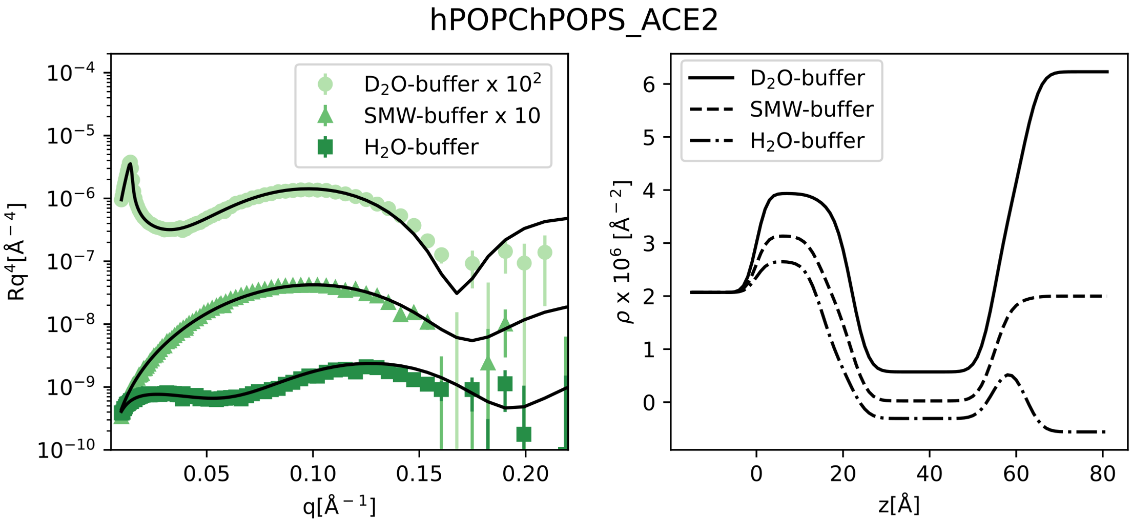

Figure 1: NR experimental data together with the fitting curves (a). Data and fits are scaled for visualisation. Scattering length density ($\rho(z)$) profile calculated from NR data analysis (b).

3 dPM after the injection of sACE2 and RDB

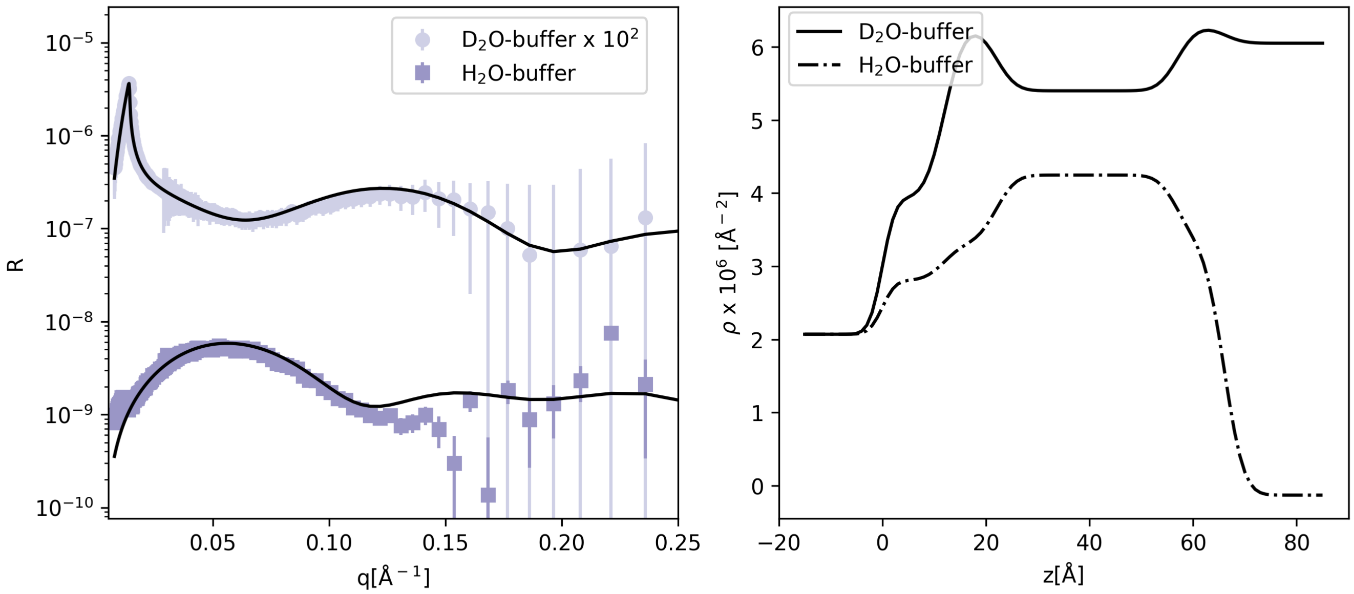

Figure 2: NR experimental data together with the fitting curves (a). Data and fits are scaled for visualisation. Scattering length density ($\rho(z)$) profile calculated from NR data analysis (b).

Table 1: Optimised parameters from NR data analysis: thickness (t), solvent volume fraction (ϕ_s), scattering length density (ρ). Surface roughness was in the range of 3-4 $\AA$ for all the investigated samples.

**dPM**	**Acyl chains**	**Headgroups**
$t[\AA]$	34 ± 3	9 ± 3
ϕ_s	0.03 ± 0.02	0.37 ± 0.08
$\rho \cdot 10^{-6}$ $[\AA^{-2}]$	5.43 ± 0.08	6.5 ± 0.6
**dPM + RDB**	**Acyl chains**	**Headgroups**
$t[\AA]$	35 ± 3	10 ± 2
ϕ_s	0.19 ± 0.02	0.5 ± 0.1
$\rho \cdot 10^{-6}$ $[\AA^{-2}]$	5.3 ± 0.1	6.5 ± 0.7

References

[1] L. G. Parratt, “Surface studies of solids by total reflection of x-rays,” *Phys. Rev.*, vol. 95, pp. 359–369, Jul 1954.
